# Evaluation of systemic inflammatory biomarkers associated with high-density lipoprotein in keratoconus patients: a retrospective case-control study

**DOI:** 10.1186/s12886-025-04511-z

**Published:** 2025-12-23

**Authors:** Burçin Çakır, Muhammed Muaz Osmanoğlu, Büşra Güner Sönmezoğlu

**Affiliations:** 1 Department of Ophthalmology, Sakarya Training and Research Hospital, Sakarya, Turkey; 2 Department of Ophthalmology, Serdivan State Hospital, Sakarya, Turkey

**Keywords:** High density lipoprotein, Progressive keratoconus, Systemic inflammation, Platelet

## Abstract

**Background:**

To evaluate systemic inflammatory markers including the monocyte to high density lipoprotein cholesterol (HDL) ratio (MHR), neutrophil to HDL ratio (NHR), lymphocyte to HDL ratio (LHR), neutrophil to lymphocyte ratio (NLR), platelet to HDL ratio (PHR) in patients with keratoconus (KC). Correlations between these systemic markers and corneal topographic parameters were also investigated.

**Methods:**

Retrospective analysis of peripheral blood sample results in patients with KC (n: 103) were performed. Mean MHR, NHR, LHR, NLR, and PHR were calculated in KC patients, and age and sex matched healthy controls (n:99). Corneal topography markers were also evaluated and correlations with systemic inflammatory markers were investigated.

**Results:**

The mean PHR was significantly higher in KC group compared to control group (*p* = 0.004). Optimal PHR cut-off value for KC was calculated as 4807 with 74.8% sensitivity and 45.5% specificity. No significant correlation was found between corneal topograhy parameters and systemic inflammatory markers.

**Conclusions:**

The mean PHR value was found to be higher in patients with keratoconus and no correlation was found between corneal topography findings and systemic inflammatory markers. This study is the first to explore HDL associated systemic inflammatory markers.

**Supplementary Information:**

The online version contains supplementary material available at 10.1186/s12886-025-04511-z.

## Introduction

Keratoconus (KC) is defined as a noninflammatory, bilateral, progressive, ectatic condition in which there is conical protrusion of a thinned central cornea [1]. The etiopathogenesis is not fully understood. As well as genetic predisposition and environmental factors, in recent years, studies revealed increase in local and systemic inflammatory markers [2–5].

Especially, neutrophil/lymphocyte ratio (NLR), monocyte/high-density lipoprotein cholesterol ratio (MHR), platelet/lymphocyte ratio (PLR) and systemic immune-inflammation index (SII) were studied as indicators of systemic inflammation [3–6]. Due to its powerful anti-inflammatory and antioxidant activities, which have a direct impact on immune responses and the possibility of developing diseases, high-density lipoprotein (HDL) has been identified as a novel inflammatory biomarker [7]. HDL related inflammatory parameters including neutrophil to ratio (NHR), lymphocyte to HDL ratio (LHR), platelet to HDL ratio (PHR), and monocyte to HDL ratio (MHR) have recently been proposed as indicators of systemic inflammation [8–13]. The neutrophil to high-density lipoprotein ratio was found to be valuable for assessing the inflammatory process in Parkinson’s disease [8]. Neutrophil/high-density lipoprotein ratio, LHR, and MHRwere also investigated as indicators of inflammation in patients with schizophrenia and bipolar disorder [11, 13].

The aim of the current study was to evaluate systemic inflammatory markers including MHR, NHR, LHR, NLR, and PHR in patients with keratoconus. Correlations between these systemic markers and corneal topographic parameters including thinnest corneal thickness, posterior elevation and keratometry results were also investigated.

## Subject and methods

A retrospective analysis was conducted with KC patients (Patient Group) hospitalized and underwent corneal collagen cross-linking procedure in the Department of Ophthalmology between January 2021 and November 2024. Age- and sex-matched healthy individuals who were examined in Ophthalmology Polyclinic composed Control Group. Prior approval was taken from the Institutional Review Board (Ethical Committee of Sakarya University, Faculty of Medicine, IRB) (IRB number: E-43012747-050.04-463782-236), and informed consent was taken from each subject. This study was in compliance with the principles of the Declaration of Helsinki.

Detailed ophthalmic examination including assessment of best corrected visual acuity (BCVA), slit-lamp and fundoscopic evaluation, and corneal tomography were performed to all subjects. The exclusion criteria for both groups were additional ocular disease, previous ocular surgeries, chronic systemic diseases which may affect systemic inflammatory markers (e.g. metabolic syndromes, obesity, and infections), systemic medical therapies, allergies, alcohol and smoking addiction. Subjects who have no signs of KC and other ocular diseases in biomicroscopic examination and corneal tomography and have venous blood sample test results taken within one month were included as Control Group.

Scissoring reflex, biomicroscopic findings such as Vogt striae, Fleischer ring, or central, paracentral thinning of cornea were main several key diagnostic features and corneal tomography provided additional parameters like high keratometry readings, posterior corneal elevation, asymmetric bow tie. Galilei G4 corneal tomography was used for corneal assessment. Change in Kmax higher than 1 diopter (D) per year with decrease in BCVA was considered as progressive KC.

Flat and steep simK, thinnest corneal pachymetry (TCP), and central corneal thickness (CCT) data in micrometer obtained from corneal tomography and BCVA were evaluated in the patient group.

Venous blood sample tests which were taken after 8–10 h fasting before corneal crosslinking procedure were included in this study. Mean neutrophil, lymphocyte, monocyte, platelet, and HDL results were obtained. Mean MHR, NHR, LHR, NLR, and PHR were calculated and compared between groups.

Statistical correlation was investigated between corneal topography data and BCVA of the eye with more severe KC and systemic inflammatory markers. The eye with higher keratometric values and lower BCVA was accepted as ‘eye with more severe KC’. The correlation between corneal topography data of fellow eyes and systemic inflammatory markers was also performed.

Statistical analysis were performed by using SPSS for Windows version 26.0 (SPSS Inc., Chicago, IL, USA). The Kolmogorov-Smirnov test was used to assess the normality of the data distribution. The chi-square test was used to analyze the qualitative independent data of both groups, while the Mann-Whitney U test was used to analyze the quantitative independent data when comparing non-normally distributed data. Correlations between corneal measurements and systemic inflammatory markers were performed by using the Spearman correlation test. To show the sensitivity and specificity of the optimal PHR cutoff value in KC patients, a receiver operating characteristic (ROC) curve was created, and the area under the curve (AUC) was calculated. A p-value less than 0.05 was considered significant.

## Results

One hundred three patients with KC (54 female, 49 male) and 99 healthy individuals (50 female, 49 male) were formed the Patient and Control groups, respectively. Post-Hoc power analysis based on the available sample size (n1 = 103, n2 = 99) and observed effect size (Cohen’s d = 0.50, medium effect) showed that the power of the study was 0.965.

No difference was observed between the two groups in terms of gender (*p* = 0.410). The mean ages of patient and control groups were 25.17 ± 5.13 and 25.40 ± 3.96, respectively (*p* = 0.707). All patients were diagnosed as progressive keratoconus.

Table 1 reveals corneal tomography data including flat and steep simK in diopter, TCP, and CCT in micron of the Patient Group.

**Table 1:** Clinical features of the eyes of keratoconus patients

	Eyes with severe KC	Fellow eyes of severe KC
Flat simK (D)	46.21±3.82	44.02±2.62
Steep simK (D)	49.99±4.42	46.36±3.47
TCP (µ)	447.33±44.43	477±39.5
CCT (µ)	467.34±45.47	493.5±39.2

KC: keratoconus, K:keratometry, TCP: thinner corneal pachymetry, CCT: central corneal pachymetry D: diopter, µ: micron

Table 2 reveals systemic inflammatory markers of both groups. The mean PHR was statistically different between groups (*p* = 0.004). After Type I error corrections, only the PHR parameter continues to show a significant difference between the patient and control groups (padj = 0.0215 for both Bonferroni and BH-FDR).

**Table 2:** Systemic inflammatory markers in both groups

	Patient Group (n:103)	Control Group (n:99)	Effect size (RBC)	%95 CI	p value
MHR	9.70±3.37	9.36±3.98	-0.11	-0.24-1.81	0.180
LHR	47.57±16.53	44.01 ± 17.02	-0.16	-1.68-8.91	0.051
PHR	5907.09±1705.56	5262.47±1504.25	-0.23	59.06-1166.06	0.004
NHR	94.68±36.97	88.28 ± 28.26	-0.07	-10.68-13.47	0.414
NLR	2.24±1.47	2.18±0.89	0.06	-0.29-0.23	0.484

MHR: monocyte/high-density lipoprotein cholesterol ratio, LHR: lymphocyte to HDL ratio PHR: platelet to HDL ratio, NHR: neutrophil to high-density lipoprotein ratio NLR: neutrophil/lymphocyte ratio, RBC: Rank-biserial correlation, CI: Confidence intervals

There were no statistically significant correlations between corneal topography indices, BCVA and systemic inflammatory markers. Correlation analysis were performed with severe and also fellow eye and no statistically significant correlation was found (Supplementary file).

**Fig. 1:**
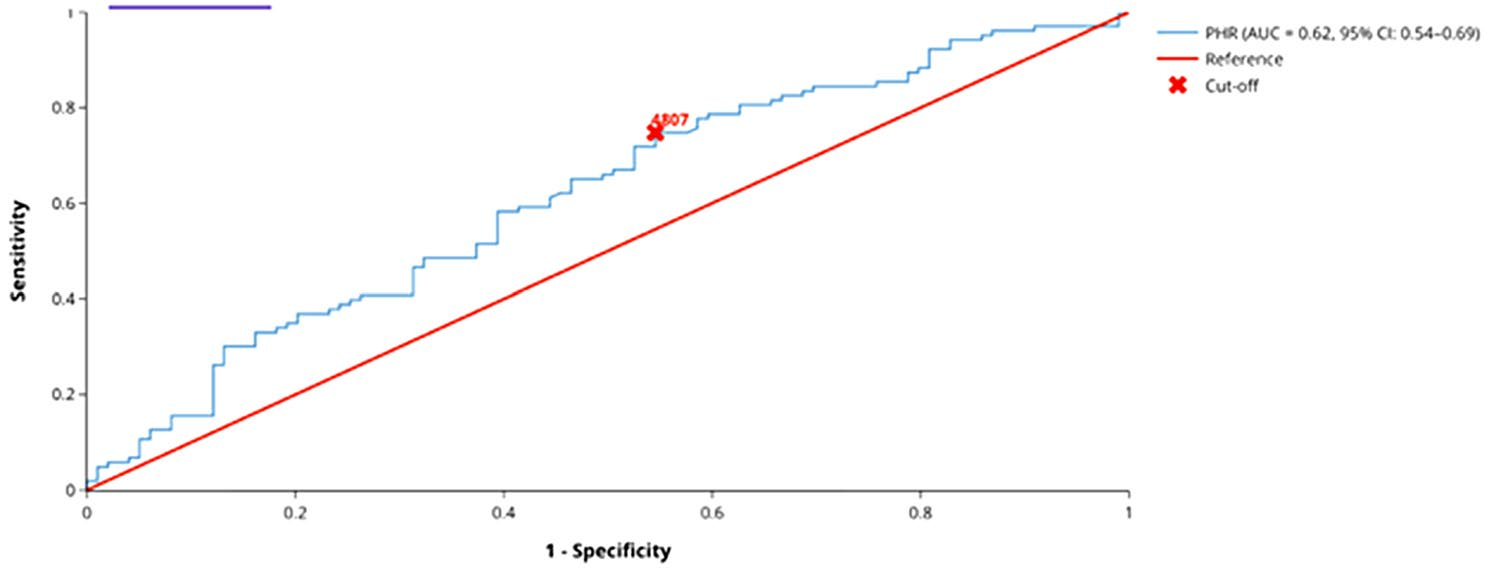
ROC analyzes for PHR

Optimal ROC cutoff value of PHR for KC was calculated as 4807 with 74.8% sensitivity and 45.5% specificity (AUC: 0.616) (Fig. 1). ROC analysis results for PHR is shown in (Tables 3).

**Table 3:** ROC analysis of PHR in keratoconus patients

	AUC	CI (95%)	SE	p value	Sensitivity(%)	Specificity(%)
PHR	0.616	0.539-0.694	0.039	0.004	74.8	45.5

PHR: platelet to HDL ratio, ROC: Receiver operating characteristic

## Discussion

In a molecular study, metabolic pathways including lipid metabolism were found to be different in keratoconus disease. According to this study, keratoconus is a condition where the cornea’s cells become damaged due to oxidative stress and lipid peroxidation. Oxidative stress stimulates the production of pro-inflammatory cytokines and enzymes, leading to cell membrane damage. Research has demonstrated alterations in antioxidant status, phosphate metabolism, and metabolites linked to the tricarboxylic acid cycle and Krebs cycle. Metabolites downregulated in keratoconus include saturated and unsaturated fatty acids, which play crucial roles in tissue repair and proliferation. These metabolites are associated with energy production, lipid metabolism, and response to oxidative stress, inflammation, and tissue damage [14]. In recent years, studies have focused on serum lipoprotein levels and their relations with other serum inflammatory markers [4, 15, 16]. High LDL levels in patients with keratoconus suggested increased oxidative stress and high MHR levels were related to inflammation in patients with keratoconus [15, 16]. Decreased HDL levels were shown to be related to inflammation and combined markers derived from peripheral blood cell measures and biochemical assays, such as the neutrophil-to-HDL ratio, lymphocyte-to-HDL ratio, monocyte-to-HDL ratio, and platelet-to-HDL ratio are under scrutiny as potential indicators of inflammation across various diseases [17, 18]. In this current study, MHR levels were not statistically different between KC patients and controls.

On the other hand, LHR, PHR, and NHR were also evaluated in our study and PHR was found to be higher in KC patients compared to controls. Platelets play a central role in inflammation by aggregating and releasing cytokines, which can amplify inflammatory responses and HDL cholesterol, known for its anti-inflammatory and antioxidant properties, facilitates dietary cholesterol efflux through reverse cholesterol transport [19]. In recent studies, PHR levels were associated with gallstone development, depression self reported stroke and cardiovascular mortality, nonalcoholic fatty liver disease, and hypertension [19–23]. To our knowledge, there was no study evaluating PHR levels in patients with KC, ocular surface diseases, and even non of other ocular diseases. The increase in PHR suggested systemic inflammatory background in KC.

In our study, we investigated the potential of PHR in KC screening and follow-up. An AUC value of 0.616, and 74.8% sensitivity and 45.5% specificity demonstrate that PHR can not be used as a predictive value for KC. Since there was no study evaluating these parameters, further investigations are needed to enlighten this item.

Although inflammation is not included in the definition of keratoconus, local proinflammatory factors have been investigated in patients with keratoconus. Lema et al. found that IL-6 and TNF-alpha were overexpressed in the tears of eyes diagnosed with subclinical KC and KC. Furthermore, increased MMP-9 levels in eyes with KC were remarkable. In light of these results, they suggested that the pathogenesis of KC may involve chronic inflammatory events [2, 24]. In a recent meta-analysis, Zhang et al. reported keratoconus as a inflammatory disease [25]. The role of local inflammation in KC suggested possible systemic inflammation in these patients and studies focused on hemogram-related systemic inflammatory markers in KC patients have been performed [3–5]. NLR and PLR levels were found to be high in patients with keratoconus compared to the nonprogressive groups and/or controls [3–5]. On the other hand, Reyhan et al. did not find any statistical difference among patients with nonprogressive, progressive keratoconus, and controls in terms of NLR and PLR levels [6]. In this current study, NLR value was not statistically significant between groups. The disease severity and progression status might alter the results.

Correlations between BCVA, corneal tomographic data and systemic inflammatory markers were investigated in the current study and no statistically significant correlation was found. Reyhan et al. reported significant positive correlations between NLR levels and corneal topography indices in KC patients but these correlations were not moderate [6]. In our study, all patients had progressive KC. Lack of non-progressive ones limited the argument about the relationship between disease severity and these inflammatory markers. In light of these findings, we can not suggest that systemic inflammatory markers might be a biomarker for detecting keratoconus.

The major limitation of the study is the single-center retrospective manner. Systemic and ocular diseases which might affect HDL levels were investigated but lifestyle variables, diet details could not be assessed The other limitation is the lack of non-progressive KC group. On the other hand, LHR, PHR, and NHR parameters derived from blood sample tests were assessed for the first time. Further prospective studies with larger sample sizes which were focused on inflammatory cytokines and so on, should be performed in order to enlighten the role of systemic inflammation in KC development.

## Conclusion

Platelet to HDL ratio was found to be statistically higher in patients with keratoconus while lymphocyte to -HDL ratio, neutrophil-to-HDL ratio, monocyte-to-HDL ratio, and neutrophil to lymphocyte ratio were not statistically different between patients with keratoconus and controls. Systemic inflammation may indicate keratoconus, but this topic requires further evidence based on cytokine profiles. Further prospective randomized clinical trials and laboratory studies should be conducted to clarify the contribution of inflammation to the pathogenesis of keratoconus.

## Supplementary Information

Below is the link to the electronic supplementary material.

Supplementary Material 1

## Data Availability

The data that support the findings of this study are available from the corresponding authors, Büşra Güner Sönmezoğlu and Burçin Çakır, upon reasonable request.

## Abbreviations

BCVA: Best corrected visual acuity
CCT: Central corneal thickness
HDL: High density lipoprotein cholesterol
KC: Keratoconus
LHR: Lymphocyte to HDL ratio
MHR: Monocyte to HDL ratio
NHR: Neutrophil to HDL ratio
NLR: Neutrophil to lymphocyte ratio
PHR: Platelet to HDL ratio
SII: Systemic immune-inflammation index
TCP: Thinnest corneal pachymetry
