# Supplementary material for: Evaluation of systemic inflammatory biomarkers associated with high-density lipoprotein in keratoconus patients: a retrospective case-control study
Source: BMC Ophthalmol. 2025 Dec 23;25:692. doi: 10.1186/s12886-025-04511-z (PMC12729630; doi:10.1186/s12886-025-04511-z)
Supplement: Supplementary file 1 — Supplementary Material 1 [file 12886_2025_4511_MOESM1_ESM.docx]

Supplementary Material 1. Correlations between BCVA, corneal tomographic data and systemic inflammatory markers in eyes with severe KC

|  | MHR | NHR | LHR | NLR | PHR |
| --- | --- | --- | --- | --- | --- |
| Flat simK (dp) | r: 0.06  p: 0.57 | r: 0.13  p: 0.19 | r: 0.08  p:0.40 | r: 0.05  p: 0.59 | r: 0.17  p: 0.08 |
| Steep simK (dp) | r: 0.08  p: 0.40 | r: 0.16  p: 0.09 | r: 0.16  p: 0.10 | r: 0.05  p: 0.61 | r: 0.17  p: 0.08 |
| TCP (micron) | r: 0.09  p:0.36 | r:0.14  p:0.14 | r:0.06  p:0.57 | r:0.07  p:0.44 | r:0.05  p:0.66 |
| CCT (micron) | r: 0.10  p:0.30 | r: 0.17  p: 0.08 | r: 0.60  p:0.55 | r: 0.09  p: 0.34 | r: 0.06  p: 0.58 |
| BCVA | r: 0.052  p:0.638 | r:0.059  p:0.593 | r:0.028  p:0.802 | r:0.085  p:0.443 | r:-0.012  p:0.913 |

K: Keratometry TCP: Thinner corneal pachymetry CCT: Central corneal pachymetry BCVA: Best corrected visual acuity, MHR: Monocyte/high-density lipoprotein cholesterol ratio, LHR: Lymphocyte to HDL ratio PHR: Platelet to HDL ratio, NHR: Neutrophil to high-density lipoprotein ratio NLR: Neutrophil/lymphocyte ratio

Supplementary Material 2. Correlations between corneal tomographic data and systemic inflammatory markers in Correlations between corneal tomographic data and systemic inflammatory markers in fellow eyes of patients with KC.

|  | MHR | NHR | LHR | NLR | PHR |
| --- | --- | --- | --- | --- | --- |
| Flat simK (dp) | r:0.001  p:0.996 | r: -0.152  p: 0.134 | r: -0.084  p: 0.407 | r: -0.048  p: 0.638 | r: -0.165  p: 0.104 |
| Steep simK (dp) | r: 0.008  p: 0.936 | r: -0.105  p: 0.301 | r: -0.057  p: 0.574 | r: -0.06  p: 0.558 | r: -0.97  p: 0.339 |
| TCP (micron) | r: 0.095  p:0.348 | r: 0.156  p: 0.122 | r: 0.057  p: 0.574 | r:0.095  p:0.352 | r: 0.032  p: 0.751 |
| CCT (micron) | r: 0.066  p:0.514 | r: 0.162  p: 0.110 | r: 0.036  p:0.721 | r: 0.101  p: 0.319 | r: 0.023  p: 0.819 |

K: Keratometry TCP: Thinner corneal pachymetry CCT: Central corneal pachymetry, MHR: Monocyte/high-density lipoprotein cholesterol ratio, LHR: Lymphocyte to HDL ratio PHR: Platelet to HDL ratio, NHR: Neutrophil to high-density lipoprotein ratio NLR: Neutrophil/lymphocyte ratio
